# Investigating the effect of Eye Movement Desensitization and Reprocessing on pain intensity in patients with primary dysmenorrhea: a protocol for a randomized controlled trial

**DOI:** 10.1186/s13063-019-3507-0

**Published:** 2019-07-08

**Authors:** Sahar Valedi, Zainab Alimoradi, Mohammad MoradiBaglooei, Amir H. Pakpour, Mehdi Ranjbaran, Venus Chegini

**Affiliations:** 1 Students Research Committee, School of Nursing & Midwifery, Qazvin University of Medical Sciences, Bahonar blv, Qazvin, 34197-59811 Iran; 2 Social Determinants of Health Research Center, Qazvin University of Medical Sciences, Bahonar blv., Qazvin, 34197-59811 Iran; 3 School of Nursing & Midwifery, Qazvin University of Medical Sciences, Bahonar blv, Qazvin, 34197-59811 Iran; 4 Department of Nursing, School of Health and Welfare, Jönköping University, Jönköping, Sweden; 5 Department of Epidemiology, School of Public Health, Shahid Beheshti University of Medical Sciences, Tehran, Iran; 6 Obstetrics and Gynecology Department, School of Medicine, Qazvin University of Medical Sciences, Qazvin, Iran; 7 Department of Epidemiology and Biostatistics, School of Public Health, Tehran University of Medical Sciences, Tehran, Iran

**Keywords:** Primary dysmenorrhea, EMDR therapy, Pain management

## Abstract

**Background:**

Unpleasant experience with the previous menstruation can increase the sensitivity to pain which may lead to moderate to severe pain in patients with dysmenorrhea. Eye movement desensitization and reprocessing (EMDR) is a psychological method to alleviate the distress from unpleasant memories and related events and can be used for other conditions such as anxiety, depression, and chronic pain. This protocol was designed to investigate the effect of EMDR therapy on pain intensity in patients with dysmenorrhea.

**Methods/Design:**

A randomized clinical trial was designed in compliance with the Consolidated Standards of Reporting Trials (CONSORT). Female students who have moderate to severe primary dysmenorrhea (based on a visual analogue scale [VAS] score of at least 4 for two consecutive months) and who live in dormitories at Qazvin University of Medical Sciences in Qazvin, Iran will be invited to participate in the study. The total sample size will be 88 girls, who will be randomly assigned to intervention (*N* = 44) and control (N = 44) groups. EMDR therapy will be performed for the intervention group, while the control group can use sedative or other pain relife methods as their routin... There will be six treatment sessions, which will be held twice a week. The duration of each session is 30–90 min, according to the convenience of each participant. The data will be collected using the demographic characteristics questionnaire, the VAS, the Subjective Units of Anxiety or Distress Scale (SUD), and the Validity of Cognition Scale (VOC). The data on pain intensity due to primary dysmenorrhea in both groups will be collected at 1 and 2 months before the intervention (to identify eligible participants) and 1 and 2 months after the intervention (follow-ups). Data will be analyzed by using SPSS version 25 software and analysis of variance (ANOVA) with repeated measures with appropriate post hoc tests. A *P* value of less than 0.05 will be considered significant.

**Discussion:**

The results are expected to provide the information on the efficacy of EMDR therapy to manage moderate to severe pain in patients with primary dysmenorrhea.

**Ethics and dissemination:**

The research proposal is approved by the human ethics committee of Qazvin University of Medical Sciences (IR.QUMS.REC.1397.100). The results of this trial will be submitted for publication in a peer-reviewed research journal.

**Trial registration:**

IRCT20180823040851N1. Registered on 6, October 2018.

**Electronic supplementary material:**

The online version of this article (10.1186/s13063-019-3507-0) contains supplementary material, which is available to authorized users.

## Background

Primary dysmenorrhea or painful menstruation is the acute pelvic pain during menstruation [1] in the absence of any diagnosed pelvic disease, including endometriosis, pelvic inflammatory disease, and pelvic leiomyoma [2]. It is one of the most common disorders among women at reproductive age; reported prevalence ranges from 16% to 93% [3–6]. It is estimated that 3–40% of patients have experienced moderate to severe pain [4, 5, 7].

It is a major disruption of quality of life and significantly affects social activities among young women [8, 9]. In severe cases, dysmenorrhea can cause problems with performing daily routine activities and absence from school or the workplace [10]. About 1% of women are absent from work for 1 to 3 days each month because of severe dysmenorrhea, and about 14% of girls are absent from school each month because of menstrual cramps [11, 12]. Dysmenorrhea high costs to the health-care system [4].

It has been proposed that the pain severity of primary dysmenorrhea is determined by the increase in or unbalanced production of prostaglandins that cause uterine contractions, decrease uterine circulation, and increase the sensitivity of sensory nerves [13]. However, several other factors, including age, marital status, body mass index, familial history, and low socioeconomic status [2, 4, 8, 9, 14], can also affect the pain severity in patients with primary dysmenorrhea. In addition to the abovementioned factors, an association between certain psychological factors (including anxiety, depression, and stress) and the pain severity during dysmenorrhea has been found [15, 16]. Depression due to the impact of pain on social and occupational functions can not only reduce the response to medication but also enhance the perception of pain severity [11, 15]. Psychological distress and increased sensitivity to pain are well known to cause idiopathic pain disorders [17]. The role of pain sensitivity in patients with severe dysmenorrhea was proposed in a review by Iacovides et al. in 2015 [2]. In that review, the severity of dysmenorrhea not only was related to increased prostaglandin production but also increased sensitivity to pain in women with primary dysmenorrhea compared with healthy controls [2]. Painful menstruation, repeated on a monthly basis, can cause a central sensitivity to pain [18]. Central sensitivity is an abnormal pain mechanism in the central nervous system and can intensify the peripheral response to pain [19]. The relationship between depression, anxiety, and stress and dysmenorrhea can also be due to the association of these factors with the increased sensitivity to pain in women with dysmenorrhea [2]. On the other hand, it can be developed because of the emotional experience of menstrual pain since pain is not just a sensory experience but can be an emotional event affecting the development of psychological distress and emotional conditions, including mood disorders [20]. Epidemiological studies and clinical literature support the hypothesis that the repetition of uterine inflammation in menstrual cycles may increase the sensitivity to pain [21].

Despite various available therapies for the treatment of primary dysmenorrhea, the pain in some cases of primary dysmenorrhea remains uncontrolled [22] and this may be due to psychological factors. The latter can exacerbate pain and other problems caused by primary dysmenorrhea in young women and girls [23]. The prevalence of depression and anxiety in women with pelvic complaints suggests the involvement of psychological mechanisms in the development of menstrual pain in addition to the physiological mechanisms [24]. Given the potential role of psychological disturbances (such as previous unpleasant experiences with menstruation) and the significant correlation between primary dysmenorrhea and anxiety, stress, and depression, the effects of using psychological methods and behavioral interventions to manage this condition should be investigated [25].

Using psychological methods and behavioral interventions to manage physiological disorders is based on the theory that both psychological and environmental factors can impact on physiological processes. The purpose of these interventions is to modify an individual’s behaviors by increasing the awareness and adaptation of certain behavior but includes interventions for modifying thoughts or recognizing individuals and changing their behavior [25, 26]. The results from a systematic review showed positive outcomes of using behavioral interventions, including biofeedback, desensitization, and relaxation for pain relief in patients with dysmenorrhea [25].

Eye movement desensitization and reprocessing (EMDR) is one of the desensitization methods which does not rely on speech therapy or drug therapy but focuses on the regular and rapid movements of the eyes [27, 28]. It is a systematic, comprehensive, non-invasive, simple, and evidence-based treatment for unpleasant memories and related events [29]. EMDR therapy has been used to treat anxiety, depression, chronic pain, phantom limb pain, somatization syndrome, and post-traumatic stress disorder [30–35]. Various studies have shown a positive effect of EMDR therapy on eliminating physical and psychological symptoms caused by unpleasant life experiences [27]. Given that most studies using this method have been performed in patients with post-traumatic stress disorder, the efficacy of this method in treating other conditions still needs further investigation [36].

Primary dysmenorrhea has a chronic nature [37] which may lead to increased sensitivity to pain due to monthly recurrent uterine inflammation during menstrual cycles [21], trauma caused by the previous unpleasant experience with menstruation [38], and anxiety and depression associated with severe dysmenorrhea. Therefore, the present study was designed to investigate the effect of EMDR on pain intensity in patients with primary dysmenorrhea.

### Research aims and hypothesis

The hypothesis is that EMDR therapy can decrease the severity of pain or pain perception in patients with primary dysmenorrhea. The aims are to investigate the effects of EMDR therapy on the intensity of pain, distress level, and validity of cognition.

## Methods

### Design and setting

**Fig. 1:**
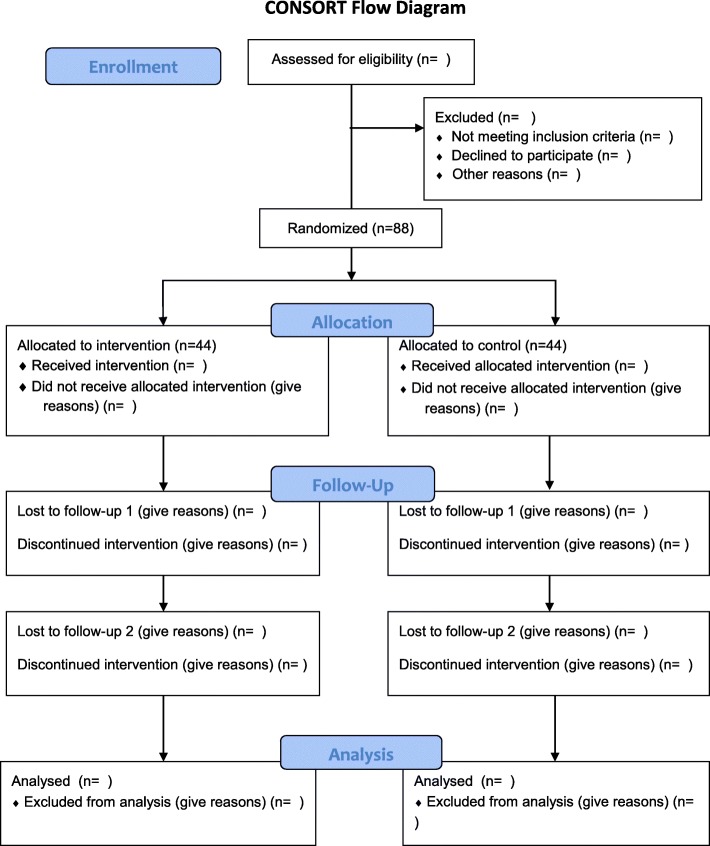
CONSORT (Consolidated Standards of Reporting Trials) flow diagram

This is a randomized clinical trial. The study design is in compliance with the Consolidated Standards of Reporting Trials (CONSORT). Female students living in dormitories at Qazvin University of Medical Sciences in Qazvin, Iran are the study subjects. Figure 1 provides the CONSORT flow diagram. The protocol of this study is prepared based on Standard Protocol Items: Recommendations for Interventional Trials (Additional file 1).

### Participants

Single female students of reproductive age (18–35 years) who live in dormitories at Qazvin University of Medical Sciences and who experience moderate to severe dysmenorrhea in most menstrual cycles will be invited to participate in this study. Exclusion criteria are (1) secondary dysmenorrhea caused by endometriosis, adenomyosis, subacute endometritis, pelvic inflammatory diseases, ovarian cysts, or congenital anomalies of the pelvis and cervix; (2) history of psychological illnesses; (3) addiction to drugs; (4) history of seizure; (5) having strabismus and vision problems; (6) history of pelvic or abdominal surgery; (7) history of cardiovascular and pulmonary respiratory disease; and (8) graduation during the follow-up period.

After the preliminary screening, potential participants will be asked to evaluate their pain intensity for two consecutive months by using the visual analogue scale (VAS). Participants who report their dysmenorrhea as moderate to severe (VAS score of at least 4) for two consecutive months will be recruited for this study.

### Intervention

EMDR therapy will be performed in the intervention group. The participants are expected to attend all scheduled interventions in which the EMDR therapy will be implemented to manage their distressing memories of severe pain due to dysmenorrhea. The expected outcome is that the level of subjective distress will be reduced and positive cognition will be reorganized [27]. The EMDR therapy consists of eight essential phases, and several steps may be performed during one session:

Phase 1

This includes taking the history of the unpleasant condition, designing the treatment protocol, and preparing and evaluating the participant. This helps the therapist to determine the specific target to achieve. To identify this target, participants will be asked to talk about their memories of the menstruation, dysmenorrhea, and menstruation-related complaints as well as the epicenter of the pain. Then they will be asked to specify the traumatic event which disturbed them the most.

Phase 2: Preparation

Prior to initiation of the intervention for the first time, therapeutic targets should be established. The procedure of EMDR therapy and its effects and safety should be explained to the participants. Their questions and concerns should be addressed.

Phase 3: Assessment

In this phase, the components of the therapeutic target should be identified. When the memory has been identified, the participant will be asked to select the image that best stands for that memory. Then the participant will be asked to choose a negative cognition that expresses the dysfunctional self-assessment related to the unpleasant condition. In this phase, negative cognition about dysmenorrhea will be identified and the participant will be asked to imagine the worst picture about their dysmenorrhea. Then they will be asked to choose a positive cognition to be used to replace the negative cognition during the installation phase. They are encouraged to imagine having a less painful menstruation as a positive cognition.

Phase 4: Desensitization

This is the desensitization step that targets the thrilling feeling of the participant. In this phase, the participants will be asked to provide their pre-treatment Subjective Units of Anxiety or Distress Scale (SUD) and Validity of Cognition Scale (VOC) scores. Then eye movement sets will be used and the distressing memory of dysmenorrhea will be processed. At the end of the phase, the client reports their level of negative affect by using the SUD.

Phase 5: Installation

This phase focuses on cognitive rehabilitation and reconstruction and on strengthening the positive cognition to replace the negative cognition. In this phase, the researcher will try to install the positive cognition of menstruation without dysmenorrhea in the participant’s mind. The participants will be asked to provide their VOC scores.

Phase 6: Body scan

This phase assesses and evaluates the physical tensions. This step is also called the physical scan. After the positive cognition is installed, the participants will be asked to hold in their mind both the target event (dysmenorrhea) and the positive cognition (menstruation without dysmenorrhea) and then scan their own body mentally from top to bottom. Then they will be asked to identify any remaining discomfort in the form of body sensation.

Phase 7: Closure

The completion or final step consists of interactive reporting and is designed primarily to maintain the balance of the participant in the session and re-evaluate her at the end. In this phase, the participants will reach a state of emotional equilibrium. They will be informed of the possibility of having disturbing images, thoughts, or emotions between the sessions. This is a positive sign and they should not be concerned.

Phase 8: Re-evaluation

This is a reassessment step and its goal is to ensure that all relevant events have been processed [39, 40].

The EMDR therapy would be performed by one of the researchers (SV). She has been trained by a clinical psychotherapist to implement this type of therapy.

### Number of intervention sessions

The number of intervention sessions was determined based on previous studies that used EMDR therapy for chronic pain management. A systematic review on the effect of EMDR therapy on chronic pain showed that 1–12 sessions (average of 6 sessions) were used to treat chronic pain. Longer sessions were used in cases of longer-term chronic pain [31]. As a result, the number of treatment sessions for this study has been set at 6, which will be held twice a week. The duration of each session is 30–90 min; this is based on the participants’ willingness and convenience [41]. Given that some participants may respond to the intervention and achieve the target before completing all of the sessions, the intervention will be continued for one more session. Then it will be documented in the final report. No intervention will be performed for the control group, but they will be asked to report their routine strategies to relieve menstrual pain.

### Measures

In this research, four instruments will be used for data collection:

*The demographic characteristics questionnaire* will be used to collect demographic and reproductive characteristics, including age, menarche age, education level, field of study, every menstrual cycle (length of menstrual period and bleeding period), and medication/drug use history. This questionnaire has been developed on the basis of the objectives of the study and its validity will be examined by the content validity method.

*The VAS* will be used to evaluate the severity of dysmenorrhea pain. An Iranian systematic review confirmed the validity and reliability of VAS for evaluation of dysmenorrheal pain [42]. The VAS is a 100-mm line where the “0” end (0) means completely painless and the other end (10) means the most intense pain imaginable. The patient marks the severity of pain on the line. The simplicity makes it easy to use for the assessment of pain [43].

*SUD* is a self-reporting scale with a 0- to 10-point rating scale. Participants are asked to report their level of distress or anxiety based on SUD scale. A score of 0 indicates the absence of distress, and a score of 10 indicates the maximum level of distress [39]. This scale has been widely used by Iranian researchers and its validity and reliability have been confirmed in their studies [44–46].

*The VOC* shows the level of belief in positive or negative recognition. Similar to the SUD scale, it is self-reporting and represents an individual’s assessment of her or his own cognition. A 7-point rating scale ranging from 1 to 7 is used to evaluate, quantify, and indicate the participant’s self-recognition. A score of 1 means a lack of belief in that particular cognition, and a score of 7 means a full belief in it [39]. This scale has been widely used by Iranian researchers and its validity and reliability have been confirmed in their studies [44–46].

### Primary outcome

The primary outcomes are the pain intensity, anxiety, or distress in patients with primary dysmenorrhea and their cognition of the dysmenorrhea.

### Secondary outcome

The secondary outcomes are the need for pain relief during menstruation and potential side effects of EMDR therapy.

### Study procedure

After obtaining permission from the human ethics committee at Qazvin University of Medical Sciences, registering it at the Iranian Clinical Trial Registration Center, and obtaining required permissions from university authorities, the researcher will visit the female dormitories at Qazvin University of Medical Sciences to invite potential participants for this study. There are four dormitories for female students at Qazvin University of Medical Sciences. The researchers will provide the information on the purpose of the research, type of intervention, and confidentiality arrangement for the research data. The participants will be asked to sign the written consent form before enrolling in the study. The researchers also ensure the participants that they can leave the study at any time and can benefit from commonly used palliative treatments whenever they are not willing to continue to participate or when they have severe pain and discomfort related to dysmenorrhea.

During recruitment, potential participants will be initially assessed for the pain intensity related to primary dysmenorrhea monthly for 2 months. Eligible individuals will be randomly assigned to EMDR therapy and control (no intervention) groups by a simple random blocking method with four blocks. EMDR therapy will be performed in the intervention group. The follow-up evaluation of dysmenorrhea pain will be performed in both groups at 1 and 2 months after the last treatment session. The flowchart of the research procedure is presented in Fig. 1.

### Sample size calculation

According to previous studies [47, 48], for an effect size of 0.25, power of 0.80, and alpha of 0.05, the sample size is to be 82 subjects (*n* = 41 in each group) using G-Power software. Given the probability of a 10% dropout rate, 44 subjects will be included in each group. The total sample size will be 88 participants.

### Statistical analysis

Data will be analyzed by using SPSS version 25 software. First, the normal distribution is confirmed by using the Kolmogorov–Smirnov test. If the data are normally distributed, analysis of variance (ANOVA) with repeated measures will be used to compare the mean scores of pain between the groups at different time points. If this is significant, appropriate post hoc tests will be used. A *P* value of less than 0.05 will be considered significant.

### Methods to protect against bias

#### Randomization and allocation

A convenience sampling method will be used for recruitment. The researchers will recruit the female students in dormitories at Qazvin University of Medical Sciences. Eligible participants will be enrolled in the study. They will be randomly assigned to one of two groups by the simple random blocking method with four blocks.

In the block randomization method, the assigning sequence will be written before the commencement of the research. Given that two groups will be studied, four blocks will be used and one letter will be assigned to one group (A: EMDR group; B: control group). All possible modes of the four blocks will be written and numbered as below:

- 1. AABB; 2. ABAB; 3. BBAA; 4. BABA; 5. ABBA; 6. BAAB.

So, in each block, two students will be assigned to the intervention group and two will be assigned to the control group. Then, with a simple random method and the table of random numbers, a number of blocks will be selected from the blocks of numbers. By writing the contents of the blocks for those numbers (as long as the sample size is determined), we will determine the sequence of random allocation. For example, if the numbers are 1, 2, 2, 3, the assigning sequence will be AABB ABAB ABAB BBAA. After the random allocation sequence is generated, it will be numbered from 1 to 88. As a result, every participant will have a unique code.

#### Allocation concealment

For the purpose of allocation concealment, the name of each group will be written on a card and placed in opaque envelopes; thereby, 88 envelopes will be prepared on the basis of the predetermined sequence. The envelopes are numbered from 1 to 88. Then the envelopes are sequentially opened for every new participant to determine the group.

### Blinding

Owing to the nature of the study, it is impossible to blind either the participant or researchers.

### Treatment fidelity

One of the researchers (SV) is trained to perform EMDR therapy. Training and monitoring of her performance on implementing the intervention will be carried out by a clinical psychotherapist. To ensure that the intervention is implemented correctly, 15% of the sessions will be randomly evaluated by using the EMDR Fidelity Rating Scale [49].

### Data management

The person (SV) responsible for collecting the data will be trained to monitor the data collection process at all stages of the study. Data entry to the SPSS software will be monitored by ZA and MR.

### Ethical and safety issues

The research protocol has been reviewed and approved by the human ethics and research committee of Qazvin University of Medical Sciences (reference number: IR.QUMS.REC.1397.100). It has also been registered in the Iranian Registry of Clinical Trials (IRCT20180823040851N1 in 2018-10-06).

In addition, the following ethical considerations will be respected during the research: obtaining informed consent from the students, the voluntary nature of the participation in this research, and the ability to withdraw from the study at any time. At the beginning and end of the study, the participants will be assured that all of the collected data are completely confidential, and their identity will remain anonymous in any presentations and publications arising from this study. In addition, before the commencement of the first treatment session, the participants will be provided with a brief explanation of EDMR therapy. The research goals will be shared with them and they will be informed of the significance of their cooperation, which will help those suffering from similar thoughts and negative emotions due to dysmenorrhea. The criteria for performing the clinical trial will be considered in the research. Furthermore, EMDR therapy will be freely available to the participants in the control group on request after the completion of this study.

## Discussion

The present study will be the first randomized clinical trial to investigate the effect of EMDR therapy on pain management in patients with primary dysmenorrhea. The results are expected to provide insight into the safety and efficacy of EMDR therapy in treating primary dysmenorrhea. The strength of the study is its design as a randomized method with good sample size. However, owing to the nature of the intervention, blinding is impossible in this study.

EMDR therapy is a systematic, comprehensive, non-invasive, simple, and evidence-based treatment for unpleasant memories and related events [29]. EMDR therapy has been used to treat various conditions, including anxiety, depression, chronic pain, phantom limb pain, somatization syndrome, and post-traumatic stress disorder syndrome [30–34]. The systematic review by Tesarz et al. (2014) showed that EMDR therapy was effective, safe, and promising in treating chronic and uncomplicated pain [35]. The present study was designed on the basis of the efficacy of EMDR therapy shown in previous study in the management of a range of conditions, including chronic pain and previous unpleasant memories. In the present study, the focus is on the chronic nature of primary dysmenorrhea and the correlation between the trauma due to the initial unpleasant experience of menstruation and associated psychosomatic disturbances, such as anxiety and depression and dysmenorrhea.

### Trial status

Recruitment has not yet begun. However, all necessary permissions have been acquired. The estimated date of recruitment is October 27, 2019. The expected date for completing the recruitment is December 30, 2019.

## Additional file

Additional file 1: SPIRIT (Standard Protocol Items: Recommendations for Interventional Trials) 2013 Checklist: Recommended items to address in a clinical trial protocol and related documents. (DOC 137 kb)

## Data Availability

Analyzed data and materials will be de-identified and published.

## Abbreviations

CONSORT: Consolidated Standards of Reporting Trials
EMDR: Eye movement desensitization and reprocessing
SUD: Subjective Units of Anxiety or Distress Scale
VAS: Visual analogue scale
VOC: Validity of Cognition Scale
